# Antibiotic Resistance, Virulence Factors, Phenotyping, and Genotyping of Non-*Escherichia coli* Enterobacterales from the Gut Microbiota of Healthy Subjects

**DOI:** 10.3390/ijms21051847

**Published:** 2020-03-07

**Authors:** Alberto Amaretti, Lucia Righini, Francesco Candeliere, Eliana Musmeci, Francesca Bonvicini, Giovanna Angela Gentilomi, Maddalena Rossi, Stefano Raimondi

**Affiliations:** 1 Department of Life Sciences, University of Modena and Reggio Emilia, via Campi 103, 41125 Modena, Italy; alberto.amaretti@unimore.it (A.A.); lucia.righini@unimore.it (L.R.); francesco.candeliere@unimore.it (F.C.); eliana.musmeci@unimore.it (E.M.); maddalena.rossi@unimore.it (M.R.); 2 Biogest-Siteia, University of Modena and Reggio Emilia, Modena, Viale Amendola 2, 42122 Reggio Emilia, Italy; 3 Department of Pharmacy and Biotechnology, Alma Mater Studiorum-University of Bologna, Via Massarenti 9, 40138 Bologna, Italy; francesca.bonvicini4@unibo.it (F.B.); giovanna.gentilomi@unibo.it (G.A.G.); 4 Unit of Microbiology, Alma Mater Studiorum-University of Bologna, S. Orsola-Malpighi Hospital, Via Massarenti 9, 40138 Bologna, Italy

**Keywords:** Enterobacterales, *Klebsiella*, *Enterobacter*, *Citrobacter*, virulence, antibiotic resistance, biofilm

## Abstract

Non-*Escherichia coli* Enterobacterales (NECE) can colonize the human gut and may present virulence determinants and phenotypes that represent severe heath concerns. Most information is available for virulent NECE strains, isolated from patients with an ongoing infection, while the commensal NECE population of healthy subjects is understudied. In this study, 32 NECE strains were isolated from the feces of 20 healthy adults. 16S rRNA gene sequencing and mass spectrometry attributed the isolates to *Klebsiella pneumoniae*, *Klebsiella oxytoca*, *Enterobacter cloacae*, *Enterobacter aerogenes*, *Enterobacter kobei*, *Citrobacter freundii*, *Citrobacter amalonaticus*, *Cronobacter* sp., and *Hafnia alvei*, *Morganella morganii*, and *Serratia liquefaciens*. Multiplex PCR revealed that *K. pneumoniae* harbored virulence genes for adhesins (*mrkD*, *ycfM*, and *kpn*) and enterobactin (*entB*) and, in one case, also for yersiniabactin (*ybtS*, *irp1*, *irp2*, and *fyuA*). Virulence genes were less numerous in the other NECE species. Biofilm formation was spread across all the species, while curli and cellulose were mainly produced by *Citrobacter* and *Enterobacter*. Among the most common antibiotics, amoxicillin-clavulanic acid was the sole against which resistance was observed, only *Klebsiella* strains being susceptible. The NECE inhabiting the intestine of healthy subjects have traits that may pose a health threat, taking into account the possibility of horizontal gene transfer.

## 1. Introduction

Important enteric pathogens belong to Enterobacterales, a bacterial order within the phylum Proteobacteria. This order encompasses permanent colonizers of the human gut that, in healthy conditions, constitute minor bacterial components of the microbiota. Opportunistic Enterobacterales can persist as gut commensals without inducing any infections, as long as the microbiota is balanced and the complex and dense bacterial community prevents their overgrowth. A bloom of Enterobacterales may occur as a result of disturbance of the microbiota, yielding pathogen-mediated infections and triggering inflammatory host responses.

*Escherichia coli* is the most studied among the Enterobacterales with regards to the traits that differentiate commensalism and pathogenicity. It normally colonizes the intestine but comprises both harmless commensals and different pathogenic variants that may instigate infections in the gut or in other tissues [1,2]. Virulent strains of *E. coli* isolated from infected patients attracted most research interest [3,4], but also fecal isolates from healthy subjects and environmental strains are the target of increasing attention, aiming to determine the pathogenic potential of a wider biodiversity reservoir [5,6].

Many non-*E. coli* Enterobacterales (hereinafter referred to as NECE) that can colonize the gut (e.g., *Klebsiella*, *Enterobacter*, *Citrobacter*, and *Serratia*) also present traits that can confer them virulence and pathogenicity or phenotypes that may result in severe heath concern, such as multidrug resistance [7,8,9,10]. The greatest efforts have been carried out to describe virulent strains, generally isolated from patients with an ongoing infection, while the pathogenic potential of NECE inhabiting the gut of healthy subjects has not been thoroughly investigated with genetic and phenotypic analysis, except for some genera [9]. The research herein presented aims to fill this gap, providing a genotypic and phenotypic description of the NECE population isolated from the feces of 20 healthy adults, and to complement a previous study that described the *E. coli* population of the same cohort of subjects [5]. A set of 32 NECE strains was isolated, taxonomically classified, subjected to PFGE genotyping, and described in terms of the genotypic determinants and phenotypic traits that may confer on them potential pathogenicity or invasiveness.

Information on the genes associated to virulence is detailed especially for *Klebsiella* spp., where a number of genes associated to harmful traits were identified, such as those encoding adhesins, siderophores (e.g., enterobactin, aerobactin, yersiniabactin), protectines, or invasins (responsible for mucoid phenotype and invasiveness), and involved in allantoin metabolism [7,11]. For other NECE genera, such as *Enterobacter*, *Cronobacter*, and *Citrobacter*, the knowledge of the genetic determinants associated to virulence and invasiveness is less comprehensive and, with few exceptions (e.g., *Citrobacter koseri*), mainly acquired from better characterized pathogens [12,13,14,15].

In addition to fimbrial and afimbrial adhesins, the production of surface cellulose structures and curli favors the adhesion of Enterobacterales and can exert a significant role in enteric biofilm-related infections [16,17]. Although not directly involved in pathogenic mechanisms, the acquisition of multiple antibiotic resistances favors the success of opportunistic Enterobacterales pathogens in invasion, survival, and spread, severely complicating the containment and treatment of infections [9,18]. Therefore, the occurrence of drug resistant bacteria within a commensal population and the possibility to exchange genetic material by horizontal gene transfer may represent a major health concern.

In the present study, multiplex PCR assays were utilized to screen the NECE, isolated from the feces of healthy subjects, for the presence of 17 main virulence genes associated to those of *Klebsiella* and *E. coli* [19,20,21]. From the point of view of the phenotype, the isolates were characterized for the ability to form biofilm and to yield curli and surface cellulose, were screened for the susceptibility to the most common antibiotics and for the ability to act as recipients in conjugation experiments, and biochemical tests were performed to compare the metabolic profile.

## 2. Results

### 2.1. Counting and Isolation of NECE

**Figure 1:**
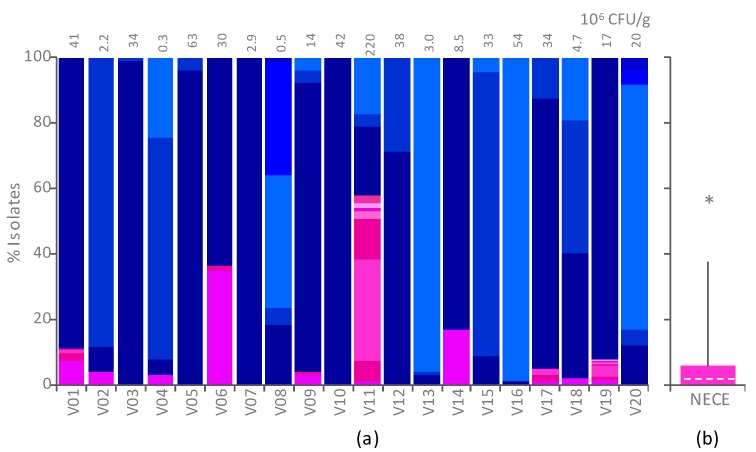
Counts of *Escherichia coli* and non-*Escherichia coli* Enterobacterales (NECE), enumerated onto HiCrome Coliform Agar (HCCA) plates. (**a**) Percentage of colonies attributed to *E. coli* (blue shades) and NECE (pink shades) in the feces of 20 subjects. For each subject, different shades indicate different biotypes according to enterobacterial repetitive intergenic consensus-PCR (ERIC-PCR) and random amplification of polymorphic DNA-PCR (RAPD-PCR) fingerprinting. The total count of Enterobacterales (*E. coli* + NECE) is reported in the top margin. (**b**) Distribution of the percentage of NECE colonies. The median (dashed line), the 25th and 75th percentiles (colored box), the 10th and 90th percentiles (whiskers), and outliers (*) are indicated.

The selective differential medium HiCrome Coliform Agar (HCCA) was utilized to count and isolate *E. coli* [5] and NECE from fecal samples of 20 healthy adults. Total counts in HCCA ranged from 4.6 × 10^5^ to 2.2 × 10^8^ cfu/g (Figure 1). Blue colonies attributed to *E. coli* overcounted the pink ones attributed to NECE in all the samples except V11 (Figure 1; Supplementary Figure S1). NECE ranged between < 10^4^ and 1.3 × 10^8^ cfu/g and, except in V11, accounted for a minority of total Enterobacterales (NECE + *E. coli*), the 75th percentile being the 5.7% (Figure 1). In some cases, NECE were not recovered, being outnumbered by *E. coli*. Spearman’s rank correlation analysis excluded any significant correlation between NECE and *E. coli* counts.

### 2.2. Taxonomic Attribution and PFGE Genotyping

The isolates putatively attributed to NECE were clustered in 32 different biotypes utilizing ERIC-PCR (enterobacterial repetitive intergenic consensus-PCR) and RAPD-PCR (random amplification of polymorphic DNA-PCR) fingerprinting. A representative isolate of each biotype was assigned a taxonomic designation utilizing 16S rRNA gene sequencing and MALDI-TOF MS (Supplementary Table S1). The genus *Klebsiella* was the most represented (14/32), with the species *K. pneumoniae* (10 strains) and *Klebsiella oxytoca* (4) found in seven and three fecal samples, respectively. The genus *Enterobacter* was represented by eight strains belonging to *Enterobacter cloacae* (6), *Enterobacter aerogenes*, and *Enterobacter kobei* (1 strain each). Other strains belonged to *Citrobacter* (4 to *Citrobacter freundii* and 1 to *Citrobacter amalonaticus*), *Cronobacter* sp. (2), and to *Hafnia alvei*, *Morganella morganii*, and *Serratia liquefaciens* (1 strain each).

**Figure 2:**
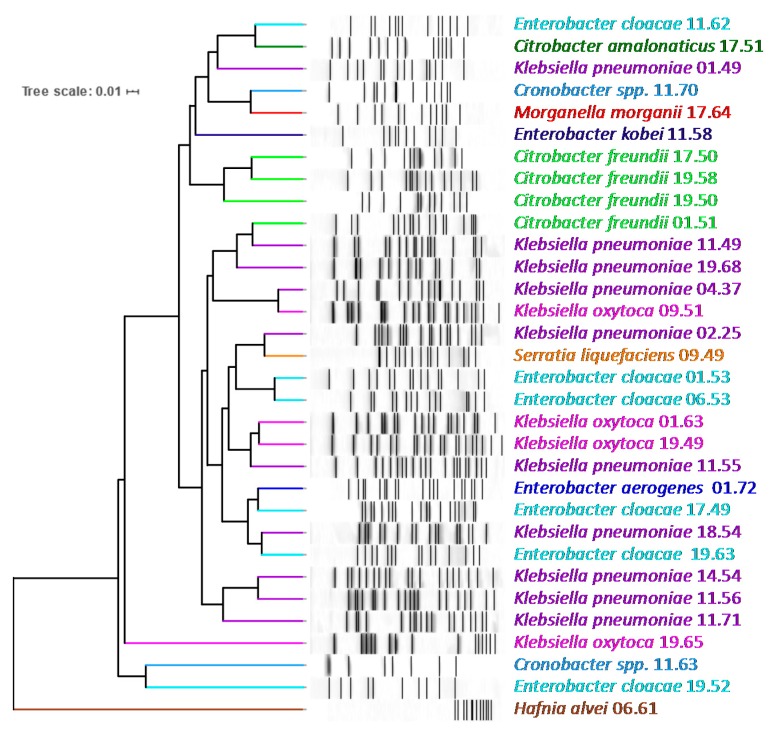
*Xba*I-PFGE pattern of NECE strains: unweighted pair group method with arithmetic means (UPGMA) dendrogram derived from Dice’s coefficients, calculated based on the band profile. Strains are colored based on their MALDI-TOF MS taxonomic attribution.

PFGE highlighted a wide diversity of the NECE isolates, which did not cluster according to the taxonomic attribution (Figure 2).

### 2.3. Virulence Genotyping

PCR was used to investigate 17 virulence genes encoding adhesins (*fimH1*, *mrkD*, *kpn*, and *ycfM*), siderophores (enterobactin, *entB*; aerobactin, *iutA*; yersiniabactin, *irp-1*, *irp2*, *ybtS*, *fyuA*; catechols receptor, *iroN*; and other, *kfu*), protectines or invasins (*K2*, *magA*, *rmpA*, and *traT*), and involved in allantoin metabolism (*allS*).

**Figure 3:**
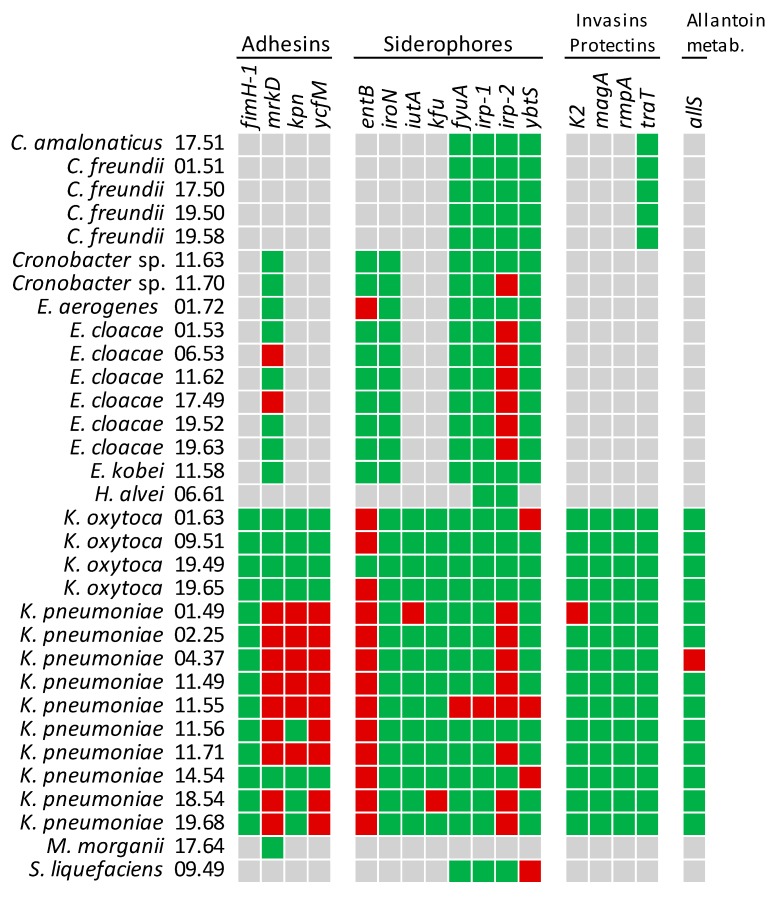
PCR assay of the NECE isolates for the presence of virulence genes. Colors: red, positive amplification; green, negative amplification; grey, PCR analysis not performed since the gene was putatively absent based on a primer-blast search.

Most strains of *K. pneumoniae* harbored the *mrkD*, *ycfM*, and *kpn* encoding adhesins and *entB* encoding enterobactin (Figure 3). Only *K. pneumoniae* 11.55 was positive to the main virulence genes involved in the synthesis of *Yersinia* siderophore, including *ybtS* (encoding for the synthase), *irp1* and *irp2* (for regulatory proteins), and *fyuA* (for the siderophore receptor). *irp2* was also detected in most of the other strains of *K. pneumoniae* although they lacked the counterpart *ybtS.* All the *K. pneumoniae* strains were negative to the genes *K2*, *magA*, and *rmpA* associated with hypermucoid phenotype and invasivity, except for *K. pneumoniae* 01.49 that was positive to *K2*. Similarly, other virulence genes, such as *allS*, *kfu*, and *iutA* occurred only once among the tested strains.

The strains of *K. oxytoca* harbored *entB* (three out of four isolates) but were negative to most of the other virulence genes. A sole strain harbored *ytbS*. Most of *Cronobacter* and *Enterobacter* isolates were characterized by the presence of the gene *irp2* but never harbored *ybtS* or other *Yersinia* siderophore genes. A few strains were positive to *mrkD* or *entB*.

The strains ascribed to *Citrobacter*, *H. alvei*, and *M. morganii* were negative to those virulence genes whose presence could not be excluded by primer-blast search. The strain of *S. liquefaciens* was positive to *ytbS*.

### 2.4. Biofilm Formation and Production of Curli and Cellulose

**Figure 4:**
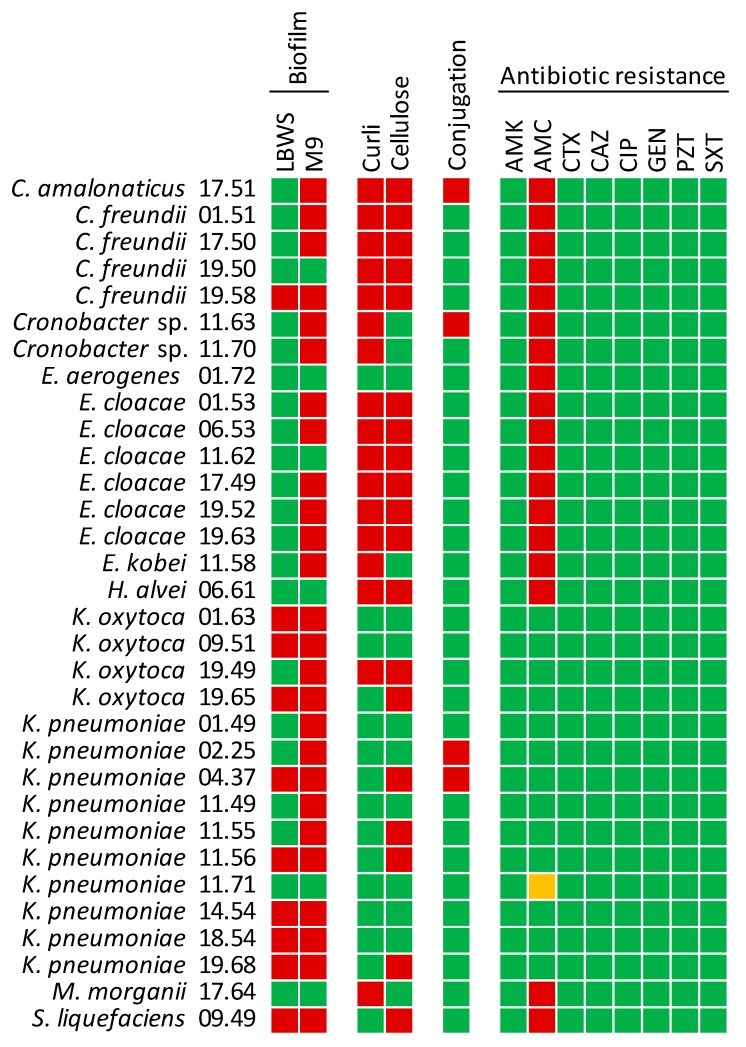
Phenotypic characterization of NECE isolates: biofilm formation in LBWS and M9 media, curli and cellulose production, conjugation, and antibiotic resistance. Colors: red, positive; green, negative. For antibiotics: red, resistant; green, susceptible, yellow, intermediate. Antibiotics: amikacin (AMK), amoxicillin–clavulanic acid (AMC), cefotaxime (CTX), ceftazidime (CAZ), ciprofloxacin (CIP), gentamicin (GEN), piperacillin-tazobactam (PZT), and trimethoprim-sulfamethoxazole (SXT).

NECE strains were tested for biofilm formation in minimal and rich media (M9 and LBWS, respectively; Supplementary Figure S2). The vast majority of the strains (26 out of 32), belonging to all the species except *E. aerogenes*, *H. alvei*, and *M. morganii*, formed biofilm in M9 (Figure 4; Supplementary Figure S2). Biofilm formation was less frequent in LBWS, being observed only in 10 strains of *K. oxytoca*, *K. pneumoniae,* and *S. liquefaciens*. The extent of biofilm production was always less abundant in the rich medium compared to M9 (*p* < 0.05).

Extracellular cellulose was detected in most of *Citrobacter* and *Enterobacter* strains, in five strains of *K. pneumoniae* and two out of four of *K. oxytoca*, in *H. alvei* and in *S. liquefaciens*. Curli were produced by nearly all *Citrobacter*, *Cronobacter*, and *Enterobacter* strains and by one isolate belonging to *K. oxytoca*. The isolates of *H. alvei* and *M. morganii* were also positive to curli. The strains belonging to *Citrobacter*, *E. cloacae*, *H. alvei,* and *K. oxytoca* 19.49 produced both cellulose and curli.

### 2.5. Conjugation

The strains were challenged as conjugation recipients for receiving pOX38: Cm plasmid from *E. coli* N4i. Only two strains of *K. pneumoniae*, and single strains of *Cronobacter* and *Citrobacter amalonaticus* succeeded in plasmid acquisition (Figure 4).

### 2.6. Antibiotic Resistance

Phenotypic susceptibility to amikacin, amoxicillin–clavulanic acid, cefotaxime, ceftazidime, ciprofloxacin, gentamicin, piperacillin-tazobactam, and trimethoprim-sulfamethoxazole was assayed. Amoxicillin-clavulanic acid was the sole antibiotic against which few isolates presented some resistance, with all the strains of *Enterobacter*, *Citrobacter*, *Cronobacter*, *H. alvei*, *M. morganii*, and *S. liquefaciens* being resistant. All the 14 biotypes of *Klebsiella* spp. were sensitive to the whole set of tested antibiotics, with the exception of *K. pneumoniae* 11.71 that was partially resistant to amoxicillin–clavulanic acid, presenting a minimum inhibitory concentration (MIC) intermediate between resistance and susceptibility thresholds.

### 2.7. Biochemical Characterization

**Figure 5:**
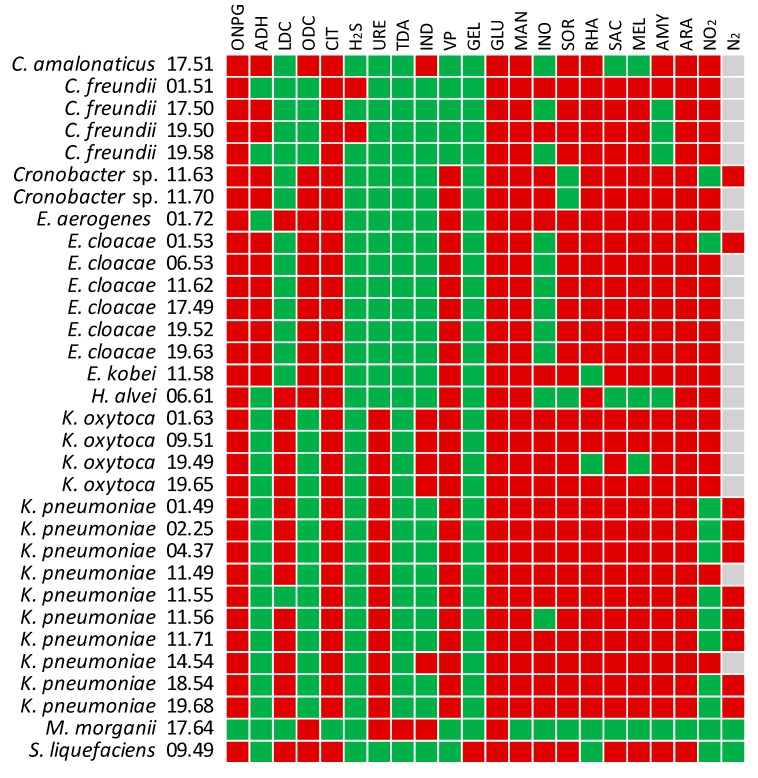
Biochemical reaction profiles of NECE isolates in the API 20 E assay: β-galactosidase (ONPG), arginine dihydrolase (ADH), lysine decarboxylase (LDC), ornithine decarboxylase (ODC), citrate utilization (CIT), production of hydrogen sulfide (H_2_S), urease (URE), tryptophan deaminase (TDA), indole (Kovac’s test, IND), acetoin (Voges-Proskauer test, VP), gelatinase (GEL), fermentation of glucose (GLU), mannitol (MAN), inositol (INO), sorbitol (SOR), rhamnose (RHA), sucrose (SAC), melibiose (MEL), amygdalin (AMY), arabinose (ARA), and reduction of nitrates to nitrites (N_2_O) or nitrogen (N_2_, tested only in case of negative N_2_O). Colors: red, positive; green, negative.

The fermentation of substrates and some distinctive enzymatic reactions and metabolic routes were assayed utilizing the API 20 E system (Figure 5). Generally, the NECE strains were positive to β-galactosidase. Most strains were capable of utilizing citrate, glucose, mannose, inositol, sorbitol, rhamnose, sucrose, melibiose, amygdalin, and arabinose. The main exceptions were *M. morganii* that could utilize only glucose, *H. alvei* that fermented a restricted number of sugars, and some *Citrobacter*, *Cronobacter*, and *Enterobacter* strains that exhibited specific substrate preferences. The majority of the isolates produced either lysine decarboxylase (*Klebsiella*) or ornithine decarboxylase (*Cronobacter* and *Enterobacter*). Urease was characteristic of *Klebsiella*, while arginine dihydrolase was found in most *Enterobacter*, *Citrobacter*, and *Cronobacter*. Acetoin was produced by *Cronobacter*, *Enterobacter*, *Hafnia,* and *Klebsiella*. Indole was produced by *K. oxytoca* and by few other strains, H_2_S by two strains of *Citrobacter freundii*. Only *S. liquefaciens* was positive to gelatinase. All the strains except *M. morganii* and *S. liquefaciens* exhibited denitrifying activity, in most cases yielding nitrite. Nitrate reduction to N_2_ was observed in *K. pneumoniae* and few other strains.

## 3. Discussion

Thirty-two NECE strains were isolated from the feces of 20 healthy adults that did not present any dysbiosis, and thus as members of a relatively balanced gut microbiota. The load of Enterobacterales was in the order of millions or tens of millions per gram of feces, with a sole exception where they reached the magnitude of 10^8^. NECE represented a small population of Enterobacterales, with *E. coli* being on average 20 times more abundant, and a minor component of the whole microbiota, being less than 0.1%. The genus *Klebsiella*, which is ubiquitous in nature, colonizing humans, animals, and plants, and frequently detected in waters, sewages, and soils, was the most represented, encompassing 14 of the 32 strains. *K. pneumoniae*, the most frequently isolated species (10 strains), was present in 7 out of 20 fecal samples, in accordance to the 35% of healthy adults from which it was isolated in a pioneering study [22].

*K. pneumoniae* encompasses opportunistic pathogens that can cause human infections in lungs, urinary tract, and bloodstreams, mostly to hospitalized and/or immunocompromised patients [23,24]. Virulence of *K. pneumoniae* is associated to the presence of capsule and pili, to the production of lipopolysaccharides and siderophores, to allantoin utilization, and to iron uptake systems, efflux pumps, and type VI secretion systems [7]. Surface molecules, such as capsular polysaccharides and lipopolysaccharides, are some of the major virulence factors that *Klebsiella* use to protect itself from the host innate immune apparatus. Furthermore, the capability to compete for iron has a pivotal role to the establishment of the infection. The genes involved in iron assimilation are generally clustered in pathogenicity islands, large chromosomal regions that were likely acquired by horizontal transfer. Virulence genetic determinants can be located both in the core or in the accessory genome [7], the former also including metabolic genes required for some species to cause disease.

Among the 10 *K. pneumoniae* isolates, all but one strain harbored *entB*, encoding a common catecholate siderophore located in the core genome, and *mrkD*, involved in the synthesis of type 3 pili that promote adherence to the surfaces [19]. The genes encoding other adhesins, such as *ycfM* and *kpn* were detected in nine and five strains, respectively. Only two *K. pneumoniae* strains were positive to the yersiniabactin gene *ybtS*, a common virulence factor associated with human infections [25,26], and one of the two also harbored *irp1*. These genes are involved in the synthesis of the siderophore yersiniabactin by virulent *Yersinia* strains, which harbor them within the high-pathogenicity island (HPI). HPI is widely distributed among members of the order Enterobacterales, including *E. coli, K. pneumoniae*, *Citrobacter* spp., *Salmonella enterica*, *Serratia liquefaciens*, and *Enterobacter* spp. [27,28]. In addition, *irp2*, another marker of HPI, was detected in the majority of the strains of *K. pneumonia* and *E. cloacae*, albeit they lacked the counterpart *ybtS*.

Although Enterobacterales are normally present as a low fraction of commensal bacteria in the healthy gut, their numbers can increase in the inflamed gut, and take advantage over other commensals [29]. Siderophores are major contributors of exploitative competition, since iron is an essential nutrient present in very low amounts in the gut and may play a role in virulence. Overall, *K. pneumoniae* 11.55 was the strain equipped with the broadest set of virulence genes involved in iron metabolism, including those encoding enterobactin (*entB*), yersiniabactin (*irp1*, *irp2*, and *ybtS)*, and, the sole strain among the 32 isolates, also the *Yersinia* siderophore receptor (*fyuA*).

In agreement with their commensal behavior, none of the *K. pneumoniae* strains were positive for the two genes associated with invasive infections, i.e., the mucoviscosity-associated gene *magA* and the regulator of mucoid phenotype *rmpA* [30,31], that are associated with a hypervirulent and hypermucoid phenotype. In our isolates, the presence of other genetic determinants of virulence was sporadic or quite rare. In the gut of the healthy host, the Enterobacterales resides in the outer loose mucus layer, separated from the epithelial mono-cell barrier by an inner dense mucin coat [32]. During the infection, they penetrate the mucus layer, interact with the epithelial cells, and may breach the mucosal barrier. In case these enterobacteria strains reach other tissues, biofilm formation may act as a fitness factor concurring to pathogenesis [33]. Adhesion factors and extracellular matrix components are involved in formation of biofilms [34]. All but one strain produced biofilm in minimal medium M9, whereas this phenotype was less frequent when growth occurred in the rich medium LBWS. The higher extent of biofilm formation in M9 is consistent with the fact that this mineral medium is more challenging for these bacteria. Extracellular cellulose structures, determined in five strains, were consistent with the capability to form biofilm on both media. Curli were found in a sole strain that presented also cellulose structures (19.58 CA) and produced biofilm on both the substrates.

Based on genetic and functional features, some *K. pneumoniae* isolates present a higher potential to cause infections, albeit they are present at low charge in the microbiota of healthy hosts. The link between colonization and infection by *K. pneumoniae* in hospitalized patients has been demonstrated, with robust evidence that their own microbiota is the main source of the infective strains [35,36]. Thus, potentially more virulent *K. pneumoniae* strains may take advantage of critical conditions, becoming responsible for nosocomial infections [37].

The isolates of *K. oxytoca*, another prominent pathogen that may be involved in diseases and life-threatening infections [38,39], generally encoded the siderophore gene *entB*, but were negative to most of the other virulence genes. A sole strain harbored the gene encoding the yersiniabactin siderophore. Biofilm production was a general feature of the *K. oxytoca* strains, regardless of the presence of curli or cellulose structures.

Similar considerations are valid for the other NECE strains herein described, belonging to genera that may have clinical relevance, such as *Enterobacter*, *Citrobacter*, and *Cronobacter*. The isolates were generally capable of forming biofilm and producing curli and cellulose and were negative for most virulence genes. However, unlike *Klebsiella* that shares many virulence genes with *E. coli*, the genetic determinants of virulence of these genera have not been fully disclosed.

In this study emerged that NECE isolates from feces of healthy subjects are still quite susceptible to most of the antibiotics. This is important, since any treatment of opportunistic outbreaks of NECE (e.g., in case nosocomial infections) requires antibiotics, but resistance developments would seriously curb the therapeutic options [40]. All the isolates of *Klebsiella* were sensitive to the whole set of tested antibiotics. Amoxicillin-clavulanic acid was the sole antibiotic against which was detected some resistance: the strains ascribed to the genus *Enterobacter* and the isolates belonging to *Citrobacter*, *Cronobacter*, *Hafnia alvei, Morganella morganii*, and *Serratia liquefaciens* were all resistant to this combination of antibiotics. Interestingly, Enterobacterales presented the highest increase in terms of relative abundance in a short-term amoxicillin-clavulanic acid treatment in healthy adults [41]. Some genera belonging to this family, such as *Enterobacter* and *Citrobacter*, are recognized as intrinsically resistant [42], and may take advantage to this antibiotic treatment. In general, the profile of resistance was independent by the subject of the fecal sample, but two clusters encompassing sensitive or resistant strains were sharply differentiated by taxonomy.

PFGE genotyping was carried out to evaluate the genetic similarity among the bacterial isolates of this study and highlighted a wide dispersion of the strains, regardless their taxonomic attribution and phylogenetic relationships. The spreading of the strains regardless of species or genera may be attributed to the presence of plasmids, the horizontal acquisition of additional genes from diverse species of Enterobacterales, and to the exchange of mobile elements that rapidly integrate and promote DNA shuffling, in agreement with the capability of some strains to accept DNA by conjugation from *E. coli* as a donor. However, the biochemical profiling mostly clustered the strains in species (data not shown), confirming that energy production and conservation, and lipid, amino acid, and nucleotide metabolism are part of the conserved reactome, despite the genome plasticity.

The Enterobacterales encountered in this study are generally recognized as opportunistic pathogens, with some potential capability to cause disease, on the basis of predicted virulence factors. Except for *K. pneumoniae* hypervirulent strains, NECE virulence seems more associated to the host features than to the strain traits. According to the similar results obtained for the *E. coli* isolated from the same cohort of healthy subjects, the absence of antibiotic resistance for most of the tested antibiotics does not pose a serious challenge for infection control. This highlights the stratification of antibiotic resistance distribution among healthy and hospitalized/diseased subjects, with NECE associated risk increasing with both illness and antibiotic therapy.

## 4. Materials and Methods

### 4.1. Isolation and Enumeration of Enterobacterales

Fresh fecal samples were collected from 20 healthy adult subjects who gave written informed consent regarding their participation in the study in accordance with the protocol approved by the local research ethics committee (reference number: 974/2019/SPER-UNIMO-ENTEROPOP; Comitato etico dell’Area Vasta Emilia Nord, Italy). The subjects—10 males and 10 females aged 35 to 45, following a western omnivore diet, and who had not been treated with prebiotics and/or probiotics for 1 month and antibiotics for 3 months—were enrolled among the employees of the University of Modena and Reggio Emilia and their relatives and were not in relationship with the researchers. 

Feces were homogenized (10% *w/v*) in isotonic Buffered Peptone Water (Sigma, Steinheim, Germany), then serial dilutions were spread onto plates of HiCrome Coliform Agar (HCCA, Sigma) and incubated at 37 °C. The medium differentiates *E. coli* (blue colonies) from NECE (salmon to red). For each subject, up to 48 colonies of putative NECE were picked and clustered into biotypes with ERIC-PCR [43] and RAPD-PCR [44] fingerprint presenting Pearson’s similarity > 75%. 

### 4.2. PFGE Genotyping

PFGE was performed according to PulseNet protocol (http://www.cdc.gov/pulsenet/PDF/ecoli-shigella-salmonella-pfge-protocol-508c.pdf). The genomic DNA was digested with 50 U of *Xba*I at 37 °C for 3 h. Fragments were resolved in a CHEF-DRIII apparatus (Bio-Rad, Hercules, CA, USA) using counter-clamped homogeneous electric field electrophoresis (24 h at 6.0 V/cm; initial switch time, 2.2 s; final switch time, 54.2 s). The run was digitally captured and analyzed with GelCompare II 6.0 software (Applied Maths NV, Sint-Martens-Latem, Belgium). Dice coefficient was computed to evaluate similarity between band profiles (position tolerance, 1%; optimization, 1%) and to derive an UPGMA dendrogram (unweighted pair group method with arithmetic means). Strains were ascribed to the same pulsotype if PFGE profile possessed >85% similarity. 

### 4.3. Taxonomic Attribution

A strain for each biotype was taxonomically characterized by partial sequencing of the 16S rRNA gene sequencing, utilizing primers targeting the V1-V3 portion. Primer sequences and PCR conditions were set up according to Raimondi et al. [45]. The sequences, obtained from a service provider (Eurofins Genomics, Ebersberg, Germany), were compared to those in SILVA SSU database utilizing SINA Aligner v1.2.11 (https://www.arb-silva.de/aligner/).

In addition, the MALDI-TOF MS-based biotyping was carried using the MALDI Biotyper 3.1 system (Bruker Daltonics, Bremen, Germany). Sample preparation for MALDI-TOF MS was performed as previously described with minor modifications [46]. Briefly, colonies of fresh overnight culture were placed on a MALDI sample slide (Bruker Daltonics) and dried at room temperature. The sample was then overlaid with 1 μL of matrix solution (α-cyano-4-hydroxycinnamic acid in 50% acetonitrile and 2.5% trifluoroacetic acid) and dried at room temperature. A MALDI-TOF MS measurement was performed with a Bruker MicroFlex MALDI-TOF MS (Bruker Daltonics) using FlexControl software and a *Escherichia coli* DH5α protein extract (Bruker Daltonics) was placed on the calibration spot of the sample slide for external calibration. Spectra collected in the positive-ion mode within a mass range of 2000–20,000 Da were analyzed using a Bruker Biotyper (Bruker Daltonics) automation control and the Bruker Biotyper 3.1 software and library (a database with 5627 entries). High confidence species identification was accepted, if the log(score) was ≥2.00, low confidence species identification log(score) values (≥1.70 and <2.00) were accepted if the three best matches showed the same species name. Any results with log(score)<1.70 were considered as an unacceptable identification.

### 4.4. Profiling of Virulence Genes

All the isolates were screened by multiplex-PCR for the genes associated to 17 virulence factors: *allS*, *entB*, *fimH*, *fyuA*, *iroN*, *irp1*, *irp2*, *iutA*, *K2*, *kfu*, *kpn*, *magA*, *mrkD*, *rmpA*, *traT*, *ybtS*, and *ycfM*. Primer sequences and amplification conditions were set up according to El Fertas-Aissani et al. [19], Compain et al. [20], and Johnson et al. [21]. In order to assess the possibility to obtain the amplicon in the different NECEs species, a search with the primer-blast tool (https://www.ncbi.nlm.nih.gov/tools/primer-blast/) was performed for all the set of primers developed for *K. pneumoniae* and *E. coli*. The result of PCR amplification was reported only for the species in which the annealing and the possibility to yield an amplicon were predicted.

### 4.5. Biofilm and Phenotype Assays

Biofilm formation was quantified with crystal violet in the microtiter assay described in [47]. Two growth media were compared: Luria Bertani without salt (LBWS) and M9 (BD Difco, Sparks, MD, USA) containing 4 g/L glucose and 0.25 g/L yeast extract. Strains exhibiting a specific biofilm formation (i.e., the ratio between crystal violet absorbance at 570 nm and culture turbidity at 600 nm) > 1. The data herein reported are the means of three independent experiments, each carried out in triplicate.

The strains were screened for curli and cellulose production utilizing LBWS agar plates supplemented with the appropriate stain [48]. Red colonies in Congo red-supplemented plates were considered positive to curli. Colonies in calcofluor white-supplemented plates that emitted fluorescence due to UV exposure (315–400 nm) were considered positive to cellulose. 

### 4.6. Solid Mating Conjugation Experiments

The strains were screened as recipients in conjugation experiments with the donor *E. coli* N4i pOX38:Cm (N4i: EcN immE7 Gmr; pOX38:Cm: Tra+ RepFIA+ Cmr) [5]. The donor and the recipient strains were cultured and put in contact onto LB plates under the conditions described in [5]. HCCA and HCCA with 20 µg/mL chloramphenicol were utilized to differentiate recipient, transconjugant, and donor colonies [5]. 

### 4.7. Antibiotic Susceptibility

The strains were tested for antimicrobial susceptibility with a Vitek2 semi-automated system (bioMerieux, Marcy-l’Étoile, France). Minimum inhibitory concentrations (MICs) were interpreted according to EUCAST (European Committee on Antimicrobial Susceptibility Testing—www.eucast.org) and susceptibility (S) or resistance (R) were defined based on the following thresholds (mg/L): amikacin, S < 8 and R > 16; amoxicillin/clavulanic acid, S < 8 and R > 8; cefotaxime, S < 8 and R > 2; ceftazidime, S < 8 and R > 8; ciprofloxacin, S < 0.5 and R > 1; gentamicin, S < 2 and R > 4; piperacillin + tazobactam, S < 8 and R > 16; trimethoprim/sulfamethoxazole, S < 40 and R > 80.

### 4.8. Biochemical Characterization

The strains were tested for distinctive enzymatic reactions and metabolic routes utilizing API 20 E test system (bioMerieux, France), according to the manufacturer’s instructions.

## Supplementary Materials

Supplementary materials can be found at https://www.mdpi.com/1422-0067/21/5/1847/s1.

## Abbreviations

NECE	non-*E. coli* Enterobacterales
HCCA	HiCrome Coliform Agar
MALDI-TOF MS	Matrix-assisted laser desorption ionization–time of flight mass spectrometry
ERIC	Enterobacterial repetitive intergenic consensus
RAPD	Random Amplification of Polymorphic DNA
UPGMA	Unweighted pair group method with arithmetic means
