# Supplementary material for: Antibiotic Resistance, Virulence Factors, Phenotyping, and Genotyping of Non-Escherichia coli Enterobacterales from the Gut Microbiota of Healthy Subjects
Source: Int J Mol Sci. 2020 Mar 7;21(5):1847. doi: 10.3390/ijms21051847 (PMC7084377; doi:10.3390/ijms21051847)
Supplement: Supplementary file 1 [file ijms-21-01847-s001.pdf]

Supplementary Table S1. Strains of NECE isolated from the feces of 20 healthy subjects. For each strain, the taxonomic attribution by both MALDI and 16S rRNA partial sequencing, and the biotype based on ERIC-PCR and RAPD-PCR fingerprinting is reported.

Strain[1]	MALDI	16S rRNA	ERIC-PCR	RAPD-PCR
17.51	*Citrobacter amalonaticus*	*Citrobacter*	E27	R25
01.51	*Citrobacter freundii*	*Citrobacter*	E26	R24
17.50	*Citrobacter freundii*	Enterobacteriaceae	E23	R21
19.50	*Citrobacter freundii*	*Citrobacter*	E24	R22
19.58	*Citrobacter freundii*	*Citrobacter*	E25	R23
11.63	*Cronobacter* spp. (*Ent. cloacae*)	*Cronobacter*	E30	R28
11.70	*Cronobacter* spp. (*Ent. cloacae*)	*Cronobacter*	E31	R29
01.72	*Enterobacter aerogenes*	*Raoultella*	E33	R31
01.53	*Enterobacter cloacae*	*Enterobacter*	E22	R12
06.53	*Enterobacter cloacae*	*Enterobacter*	E15	R14
11.62	*Enterobacter cloacae*	*Enterobacter*	E16	R15
17.49	*Enterobacter cloacae*	*Enterobacter*	E20	R19
19.52	*Enterobacter cloacae*	*Enterobacter*	E21	R20
19.63	*Enterobacter cloacae*	*Enterobacter*	E17	R16
11.58	*Enterobacter kobei*	Enterobacteriaceae	E19	R18
06.61	*Hafnia alvei*	*Hafnia-Obesumbacterium*	E28	R26
01.63	*Klebsiella oxytoca*	*Klebsiella*	E14	R13
09.51	*Klebsiella oxytoca*	Enterobacteriaceae	E11	R10
19.49	*Klebsiella oxytoca*	*Klebsiella*	E13	R12
19.65	*Klebsiella oxytoca*	*Klebsiella*	E12	R11
01.49	*Klebsiella pneumoniae*	*Klebsiella*	E10	R09
02.25	*Klebsiella pneumoniae*	*Klebsiella*	E01	R01
04.37	*Klebsiella pneumoniae*	*Klebsiella*	E02	R02
11.49	*Klebsiella pneumoniae*	*Klebsiella*	E03	R03
11.55	*Klebsiella pneumoniae*	*Klebsiella*	E06	R05
11.56	*Klebsiella pneumoniae*	*Klebsiella*	E04	R04
11.71	*Klebsiella pneumoniae*	*Klebsiella*	E05	R01
14.54	*Klebsiella pneumoniae*	Enterobacteriaceae	E07	R06
18.54	*Klebsiella pneumoniae*	*Klebsiella*	E08	R07
19.68	*Klebsiella pneumoniae*	*Klebsiella*	E09	R08
17.64	*Morganella morganii*	*Morganella*	E32	R30
09.49	*Serratia liquefaciens*	*Serratia*	E18	R17

[1] Strains are numbered as xx.yy, where xx and yy represent the subject and the colony ID, respectively.

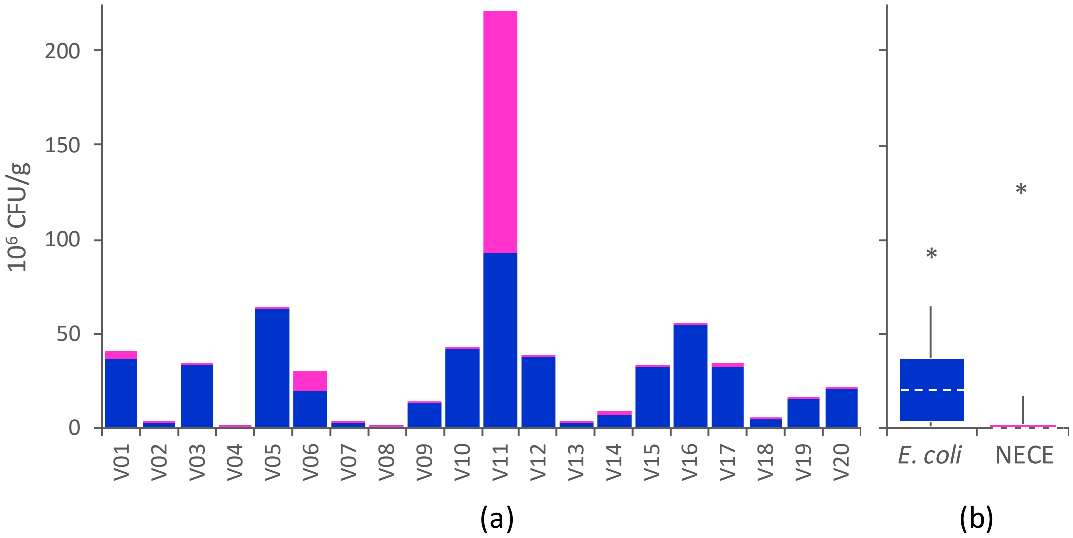

Supplementary Figure S1. (**a**) Plate counts of *E. coli* (blue) and NECE (pink) in the feces of 20 subjects. (**b**) Distribution of *E. coli* and NECE colonies. The median (dashed line), the 25th and 75th percentiles (colored box), the 10th and 90th percentiles (whiskers), and outliers (*) are indicated.

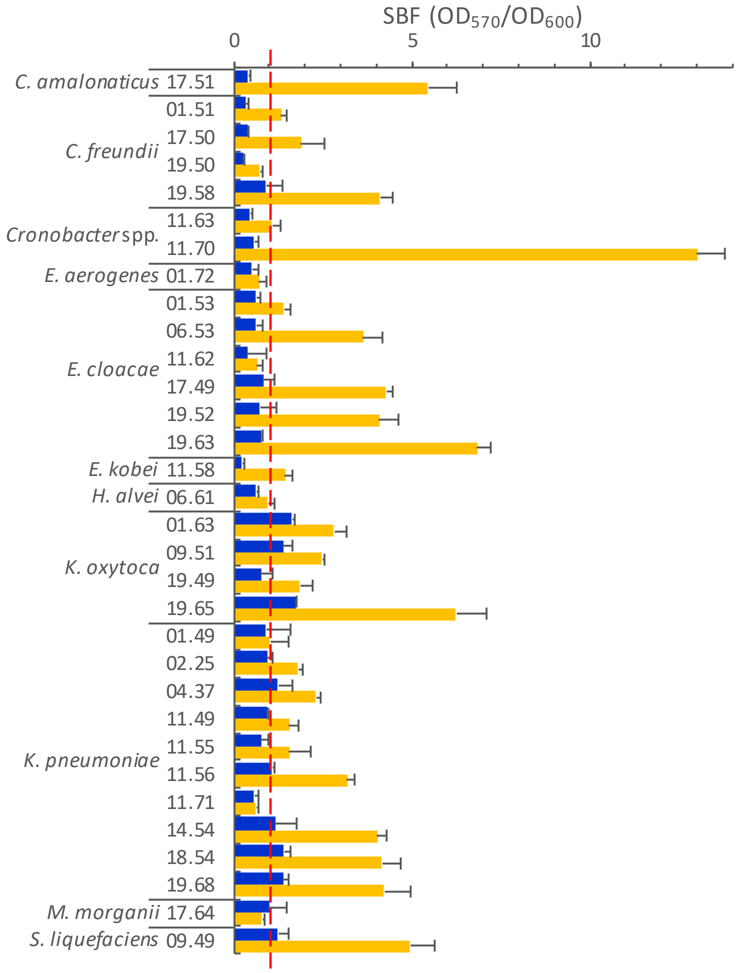

Supplementary Figure S2. Specific biofilm formation (SBF) of NECE strains in M9 (yellow) and LBWS (blue media). The reported data are means ± standard deviations of at least three independent experiments, each carried out in triplicate.
